# Effect of Osteoblast‐Specific Deletion of the Proton Receptor OGR1

**DOI:** 10.1002/jbm4.10691

**Published:** 2022-10-31

**Authors:** Nancy S Krieger, Luojing Chen, Jennifer Becker, Michaela Chan, David A Bushinsky

**Affiliations:** ^1^ Division of Nephrology, Department of Medicine University of Rochester School of Medicine and Dentistry Rochester NY USA

**Keywords:** ACIDOSIS, GENETIC ANIMAL MODEL, OGR1, OSTEOBLAST

## Abstract

Metabolic acidosis (MET) stimulates bone resorption through inhibition of osteoblast (OB) bone formation and stimulation of osteoclast (OC) bone resorption. We found that OGR1, a G protein‐coupled proton (H^+^)‐sensing receptor, was critical for initial H^+^ signaling in the OB. In mice with a global deletion of OGR1, we demonstrated that loss of OGR1 impairs H^+^‐induced bone resorption, leading to increased bone density through effects on both the OB and OC. Using an OC‐specific deletion of OGR1, we found that MET directly activates OGR1 in the OC. To determine if the response of OGR1 to MET in the OB is independent of a response in OCs and to characterize direct activation of OGR1 in the OB, we studied female mice with an OB‐specific deletion of OGR1 (OB‐cKO) and differentiated osteoblasts derived from marrow of OB‐cKO and wild‐type (WT) mice. In OB‐cKO mice, we found increased bone area in both tibial and femoral cortical bone. Specific loss of OB OGR1 increased in vitro mineralization, alkaline phosphatase activity, and expression of osteoblast‐specific genes compared with WT with no alteration in OC activity. MET stimulation of OB *cox2* and *fgf23* gene expression was inhibited in OB‐cKO OB. These results indicate that MET activation of OGR1 in the OB is independent of the response in the OC and that OGR1 in both cell types is required for a complete response to MET. Characterization of the role of OGR1 in MET‐induced bone resorption will improve our understanding of bone loss associated with metabolic acidosis in patients with chronic kidney disease. © 2022 The Authors. *JBMR Plus* published by Wiley Periodicals LLC on behalf of American Society for Bone and Mineral Research.

## Introduction

Metabolic acidosis (MET) acts directly on bone, leading to a decrease in bone mineral.^(^ ^1^, ^2^, ^3^ ^)^ During MET, bone buffers the additional protons, which helps normalize systemic pH in part through resorption of calcium (Ca) and associated anions. To understand the mechanism by which MET directly regulates bone resorption, we have used a physiologic in vitro mouse model. During MET, there is an initial loss of Ca by physicochemical dissolution followed by cell‐mediated resorption first observed at 24 hours.^(^ ^3^, ^4^, ^5^, ^6^, ^7^ ^)^ We found that MET inhibits osteoblastic bone formation and stimulates osteoclastic bone resorption.^(^ ^5^ ^)^ The initial step in response to MET in the osteoblast (OB) is an increase in intracellular Ca (Ca_i_) signaling^(^ ^8^, ^9^, ^10^ ^)^ and stimulation of cyclooxygenase 2,^(^ ^11^ ^)^ leading to a prostaglandin E_2_‐mediated increase in receptor activator of nuclear factor κB ligand (RANKL) expression.^(^ ^12^, ^13^ ^)^ RANKL binds to its specific receptor on the osteoclast (OC) to promote differentiation and activation of OCs, leading to active bone resorption.^(^ ^14^, ^15^ ^)^

The ovarian cancer G protein‐coupled receptor (OGR1 or GPR68) senses extracellular protons (H^+^) through its histidine residues and is coupled to Gq, which stimulates inositol phosphate (IP_3_) production and subsequent mobilization of Ca_i_ when activated by an increase in H^+^ concentration.^(^ ^16^, ^17^ ^)^ OGR1 expression is upregulated in many neoplastic cells and may play a role in tumor biology^(^ ^16^ ^)^ and is important in the response of bone to acidosis.^(^ ^8^, ^18^, ^19^, ^20^, ^21^ ^)^ We and others have found that bone cells, including osteoblasts,^(^ ^8^, ^18^, ^19^ ^)^ osteocytes,^(^ ^18^ ^)^ and osteoclasts,^(^ ^22^, ^23^, ^24^, ^25^, ^26^ ^)^ express OGR1. Proton activation of OGR1 results in increased Ca_i_ signaling in osteoblasts, leading to increased bone resorption in cultured neonatal mouse calvariae.^(^ ^8^ ^)^ CuCl_2_, an OGR1 inhibitor that directly stabilizes histidine residues in OGR1, inhibits this resorption in cultured neonatal mouse calvariae.^(^ ^8^ ^)^ Using pharmacologic inhibition of IP_3_‐mediated Ca_i_ release, we also found inhibition of H^+^‐induced intracellular signaling in osteoblasts and bone resorption.^(^ ^10^ ^)^ These findings strongly suggest that OGR1 is the H^+^ sensor in the osteoblast that detects the increase in [H^+^] during MET and initiates osteoblastic signaling, leading to increased osteoclastic bone resorption.

Using mice with a global deletion of OGR1, we demonstrated that loss of OGR1 led to increased bone mineral density and changes in both OB and OC activity.^(^ ^20^ ^)^ More recently, we found, using an OC‐specific knockout of OGR1 (OC‐cKO), that MET can directly activate OC; specific deletion of OGR1 from osteoclasts led to increased bone density and decreased number and activity of osteoclasts.^(^ ^26^ ^)^ To determine if the response of OGR1 in the OB is independent of a response in the OC and, if so, to define the role of direct activation of OGR1 in OB by MET, we generated a conditional knockout mouse with an osteoblast‐specific deletion of OGR1 (OB‐cKO). In this study, we present the results from female knockout mice compared with wild‐type mice.

## Materials and Methods

### Generation of OB‐specific knockout mice

**Fig. 1:**
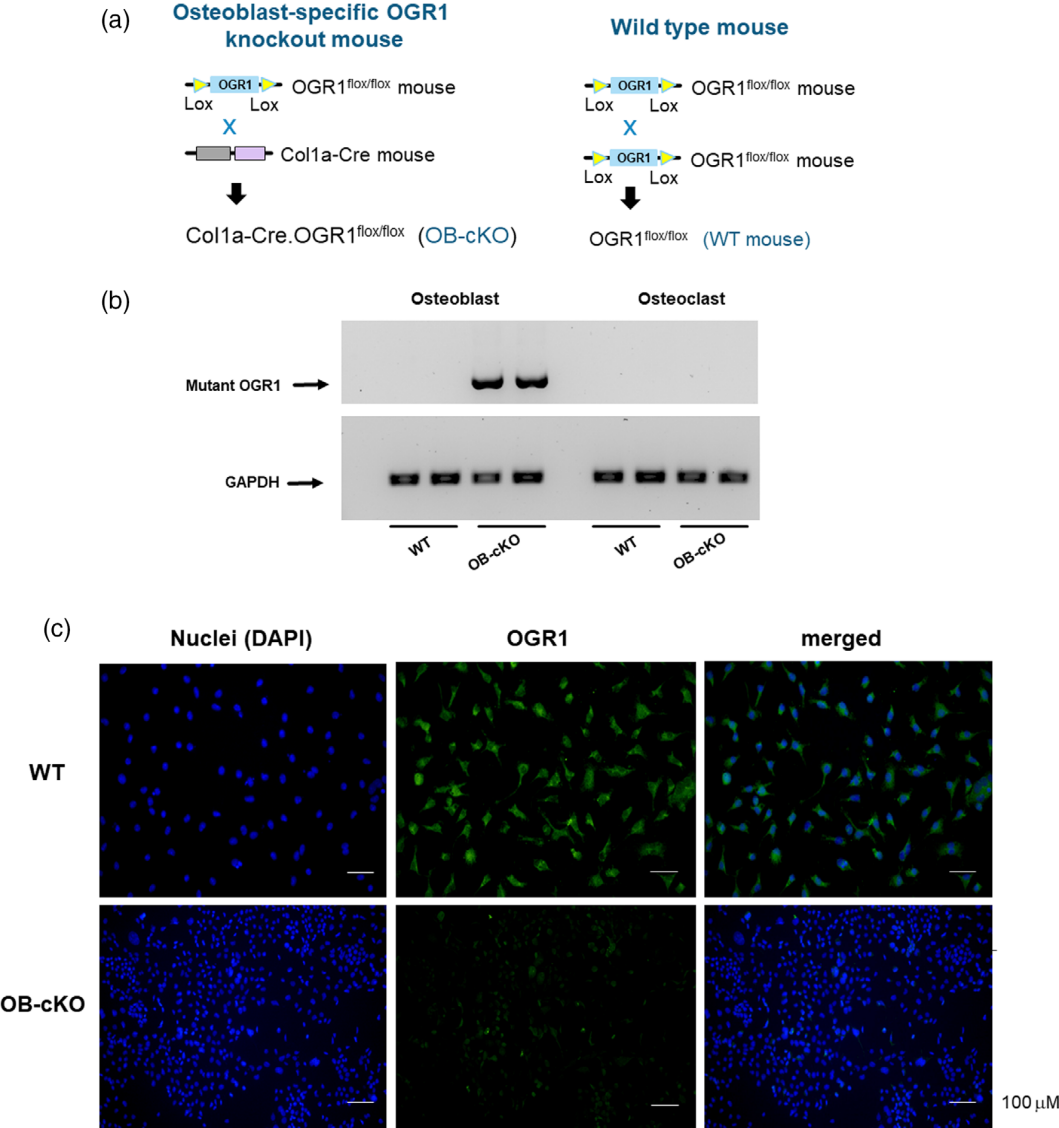
Detection of deleted OGR1. Bone marrow stromal cells (BMSCs) were obtained from wild‐type (WT) mice or conditional knockout mice with an osteoblast‐specific deletion of OGR1 (OB‐cKO) and differentiated to osteoblasts and cultured for 7 days. (*A*) Breeding scheme to generate OB‐cKO mice compared with WT mice. (*B*) DNA from BMSCs was analyzed by PCR for detection of OGR1‐specific deletion; GAPDH was used as a control. (*C*) Immunofluorescent staining of OGR1 in osteoblasts differentiated from BMSCs of WT and OB‐specific OGR1 cKO mice. Cells were stained with OGR1antibody (green) and DAPI (blue) to indicate nuclei.

OGR1‐floxed (fl/fl) mice (on a C57/Bl6j background) were generously provided by Dr Yan Xu, Indiana U School of Medicine.^(^ ^25^ ^)^ *Ogr1* flox allelic combinations were identified by standard PCR using the primers (common forward primer: 5′‐aaagcacagaaggatgcggagc‐3′; mutant flox: reverse primer 5′‐ccagcacggtcacatagaccac‐3′; wild‐type reverse: 5′‐ccagacggtcacatagaccac‐3′) on genomic DNA from tail snips. To generate OB‐specific OGR1 conditional knockout mouse, OGR1‐floxed mice were bred with Col1a1‐cre transgenic mice, also on a C57/Bl6j background (generously provided by Dr Michael Zuscik, University of Rochester School of Medicine).^(^ ^27^ ^)^ The Col1a‐Cre allele was identified by PCR using the following primers: forward 5′‐cctggaaaatgcttctgtccgtttgcc‐3′, and reverse primer 5′‐gagttgatagctggctggtggcagatg‐3′. In this system, the Cre gene is under the control of the 2.3‐kb proximal fragment of the alpha1(I)‐collagen promoter, which is expressed at very high levels in mature osteoblasts,^(^ ^27^, ^28^, ^29^ ^)^ and we estimate about 70% Cre efficiency in recombination, based on immunofluorescent staining of cultured osteoblasts for the presence of OGR1. Multiple slides were analyzed for the absence or presence of OGR1; negative staining indicated successful recombination. In this study, the mutant mice carrying the floxed *ogr1* gene on both alleles (*ogr1* ^fl/fl^) as well as Col1a‐Cre are defined as OB‐cKO, while the age‐matched mice with both ogr1 alleles (*ogr1* ^fl/fl^), but without Col1a‐cre allele, are defined as wild‐type controls (WT) (Fig. 1*A*). All mice described in this study were maintained on the C57/Bl6j background. We examined bones from 3‐month‐old female mice using micro‐computed tomography (μCT) and immunohistochemistry. All experiments were performed in accordance with guidelines of the National Institutes of Health and approved by the University of Rochester Medical Center Animal Care Committee.

### Osteoblast differentiation

Bone marrow stromal cells (BMSC) from femurs of 10‐ to 12‐week‐old female OB‐cKO and OGR1 flox/flox (WT) mice were cultured to near confluence and then differentiated to OB by incubation with 50 μg/mL ascorbic acid and 10 mM β‐glycerophosphate.^(^ ^30^ ^)^ After 1 week in differentiation medium, DNA was collected from the cells and genotyping was used to determine the presence of the mutant OGR1. As an additional control, mesenchymal stem cells from bone marrow of WT and OB‐cKO mice were differentiated to osteoclasts (see below) to determine whether OGR1 was deleted specifically only in OB cells but not in OC cells. PCR with specific primers was used to identify the OGR1 deletion in OB cells from OB‐cKO mice when compared with intact OGR1 in OB cells derived from WT mice. PCR products were analyzed on 1.5% agarose gels.

In other experiments, BMSC were cultured in differentiation medium for ≥2 weeks with medium changes every 3 to 4 days. After 2 weeks in differentiation medium, cells were washed, fixed with 10% neutral buffered formalin, and then stained with BCIP/NBT solution (Thermo Fisher Scientific, Waltham, MA, USA) to measure alkaline phosphatase or after 3 weeks were washed, fixed, and stained with alizarin red.^(^ ^30^ ^)^ Quantitation was carried out with a ChemiDoc MP imaging system (Bio‐Rad, Hercules, CA, USA). After 2 weeks in differentiation medium, cells on other plates were collected for RNA measurements of specific gene expression or used to test responsiveness to MET. In indicated experiments, medium was changed to pre‐equilibrated medium with the pH adjusted to pH = 7.45 for neutral or pH = 7.15 to model metabolic acidosis, and plates were incubated for an additional 6 hours before collecting RNA. Medium pH was adjusted by the addition of 2.4 N HCl to reduce bicarbonate concentration as a physiologic model of MET, and the partial pressure of CO_2_ was maintained at the physiologic normal of ~40 mm Hg.^(^ ^7^, ^11^, ^31^ ^)^ No other buffers were utilized to maintain medium pH.

### Osteoclast differentiation

Bone marrow hematopoietic stem cells were isolated from femurs of 10‐ to 12‐week‐old female WT and OB‐cKO mice.^(^ ^26^ ^)^ To generate OC, cells were cultured in α‐MEM containing 5 ng/mL macrophage colony‐stimulating factor (M‐CSF). After 2 days, fresh medium also containing 25 ng/mL RANKL was added and cells were cultured for an additional 4 days to induce OC differentiation. Cells in 96‐well plates were then stained for tartrate‐resistant acid phosphatase (TRAP). Dark red TRAP‐positive cells having three or more nuclei were counted as OC. In other experiments, total RNA was isolated from 100 mm plates and used for qPCR analysis of specific osteoclastic gene expression.

### Immunofluorescent staining

One week after confluence, differentiated cells were fixed with 4% paraformaldehyde, permeabilized with 0.2% Triton X‐100, and then washed, followed by staining with a primary OGR1 antibody for 45 minutes at room temperature and then with Alexa Fluor‐488‐conjugated anti‐mouse secondary antibody.^(^ ^26^ ^)^ After a final wash, nuclei were stained with the fluorescent dye DAPI (4′, 6‐diamidino‐2‐phenylindole; Sigma, St. Louis, MO, USA).

### Micro‐CT analysis

To assess bone microstructure, right femur, tibias, and L2 vertebrae were isolated from OB‐cKO and age‐matched WT female mice after euthanization using 100% CO_2_ inhalation. Bones were fixed in 10% neutral‐buffered formalin and scanned using a Viva CT 40 μCT scanner (Scanco Medical, Bruttisellen, Switzerland) to image bones at a voxel size of 10.5‐micron. Tibia cortical midshaft analysis starts at a standard shape roughly 1 mm above the lower fibular union, then proceeds 30 slices distally. Femur cortical midshaft analysis selects a standard visual shape near the distal end of the third trochanter, then proceeds 30 slices distally. The first and last slice are for support only, and 28 slices of each tibia and femur are analyzed. Contours are “snapped” directly to the cortical bone, allowing a subtraction of Total Area – Bone Area = Marrow Area. A Scanco threshold of 300 (2.40^−cm^) was used. Tibia trabecular analysis begins at the distal end of that growth plate and proceeds 100 slices (10.5 micron each) distally. The first and last slice are for support only and are not analyzed. Contours are close‐drawn to the cortical shell, and then shrunk to 95% in *x* and *y* dimensions to avoid any inclusion of cortical bone. A Scanco threshold of 280 (2.24 cm^−1^) was used. Three‐dimensional reconstructions generated from two‐dimensional images were used to calculate morphometric parameters, including bone volume/tissue volume, trabecular thickness, trabecular number, trabecular spacing, cortical bone area, mean bone mineral density, and cortical bone and trabecular bone mass.

### QPCR

To determine specific osteoblast gene expression, total RNA was isolated from cells using Qiagen RNeasy Kits (Qiagen, Hilden, Germany). The RNA was reverse transcribed into first‐strand cDNA using an oligo dT/random primer mix (iScript, Bio‐Rad). Analysis of relative changes in mRNA content was determined in a real‐time instrument (CFX Connect, Bio‐Rad) with SYBR Green 490 as fluor, using a threshold transition model, and results are normalized to *RPL13a* content to determine relative expression of each gene. Standard curves were established for each primer pair. Quantification of PCR products was performed using the comparative cycle threshold method as described previously.^(^ ^32^ ^)^

### Data analysis

Statistical analysis was performed using the Student's *t* test for unpaired comparisons or two‐way ANOVA (Fig. 8) with conventional computer programs (Statistica, StatSoft, or Prism, GraphPad, La Jolla, CA, USA). Data are presented as mean ± SEM, and *p* < 0.05 was considered significant.

## Results

### Osteoblast‐specific deletion of OGR1

Similar to the protocol we used to obtain osteoclast‐specific deletion of OGR1,^(^ ^26^ ^)^ we bred OGR1‐floxed mice (which were used as WT for all experiments) with Col1a1‐Cre mice to generate osteoblast‐specific knockout (OB‐cKO) mice. Standard tail snip genotyping was carried out for identifying OGR1 flox/flox/cre mice. To determine whether OGR1 was successfully and specifically deleted in osteoblasts, bone marrow stromal cells were cultured under conditions leading to osteoblast differentiation (see Materials and Methods), and those osteoblasts were used to determine the deletion of OGR1 with PCR and primers specific for identifying the OGR1 deletion. The OGR1 deletion mutant was only present in osteoblasts from OB‐cKO mice but not in the WT osteoblasts (Fig.1B). The OGR1 deletion was not observed in osteoclasts differentiated from either WT or OB‐cKO mice. Further support for successful deletion of functional OGR1 from OB‐cKO osteoblasts was demonstrated by immunofluorescent staining of OGR1 in differentiated osteoblasts. Intact OGR1 was present in WT osteoblasts but not in OB‐cKO cells (Fig. 1C) *1*. No gross phenotypic differences were observed between WT and OB‐cKO mice.

### Micro‐CT of bone from OB‐cKO and WT mice

**Fig. 2:**
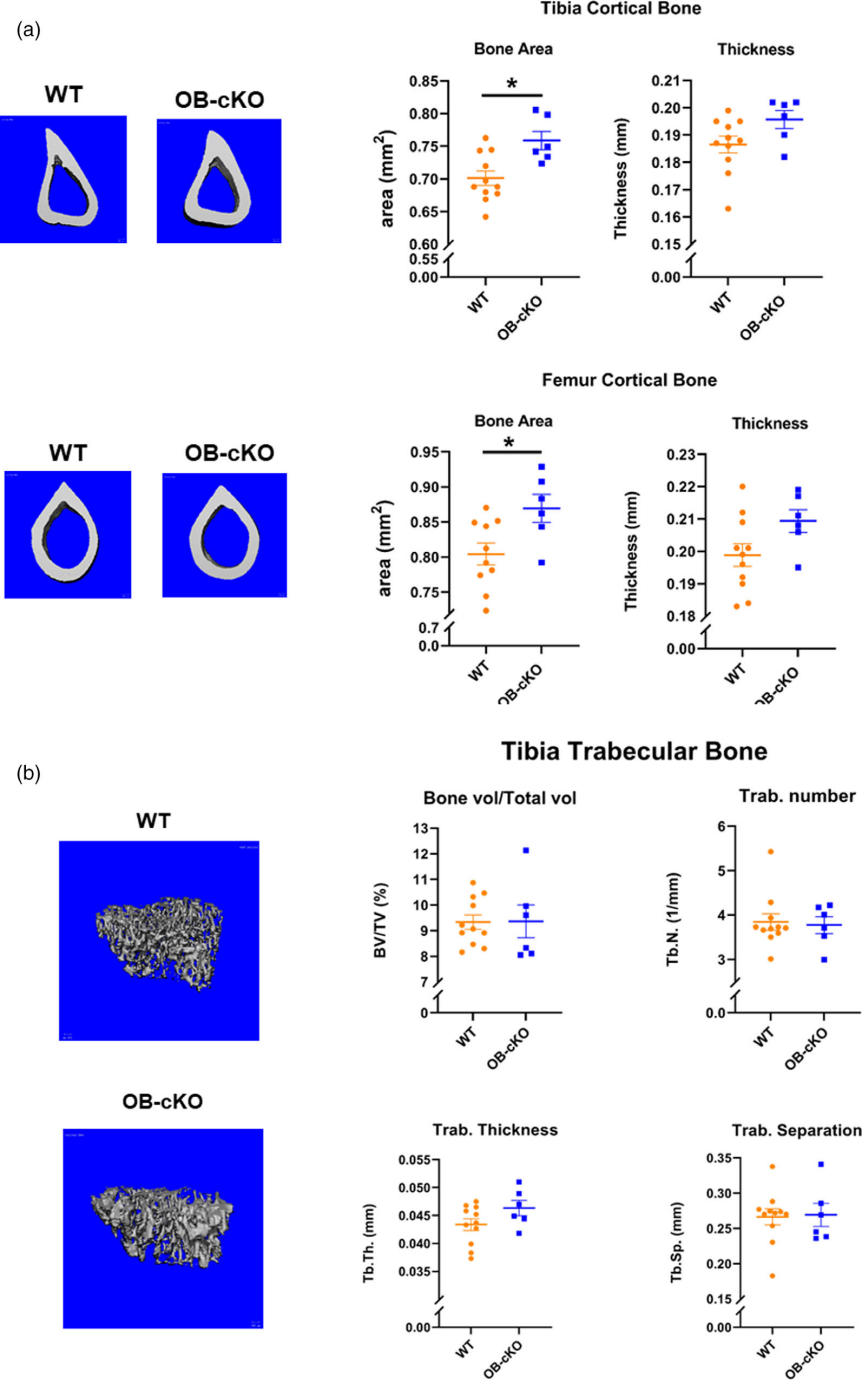
Micro‐CT of tibias and femur from 3‐month‐old female wild‐type (WT) mice and conditional knockout mice with an osteoblast‐specific deletion of OGR1 (OB‐cKO). (*A*) Left panels: representative micro‐CT images; right panels: tibial and femoral cortical area and thickness. (*B*) Left panels: representative micro‐CT images; right panel: tibia trabecular % bone volume, number, thickness, and separation. Results are mean ± SE for 6–11 mice/group. *p < 0.05 versus WT.

Micro‐CT analysis demonstrated a significant increase in bone area in tibia cortical bone and femoral cortical bone (Fig. 2*A*). Although we have observed significant differences in micro‐CT of trabecular bone in both the global OGR1 knockout male mice^(^ ^20^ ^)^ and OC‐cKO female mice,^(^ ^26^ ^)^ in these OB‐cKO mice, no differences were observed in tibia trabecular bone compared with WT mice (Fig. 2*B*) or in femoral trabecular bone (data not shown). This suggests that OGR1 in the osteoblast is important in regulation of bone formation, but OGR1 in the osteoclast is also critical to the overall bone phenotype.

### Changes in osteoblast activity in OB‐cKO cells compared with WT cells

**Fig. 3:**
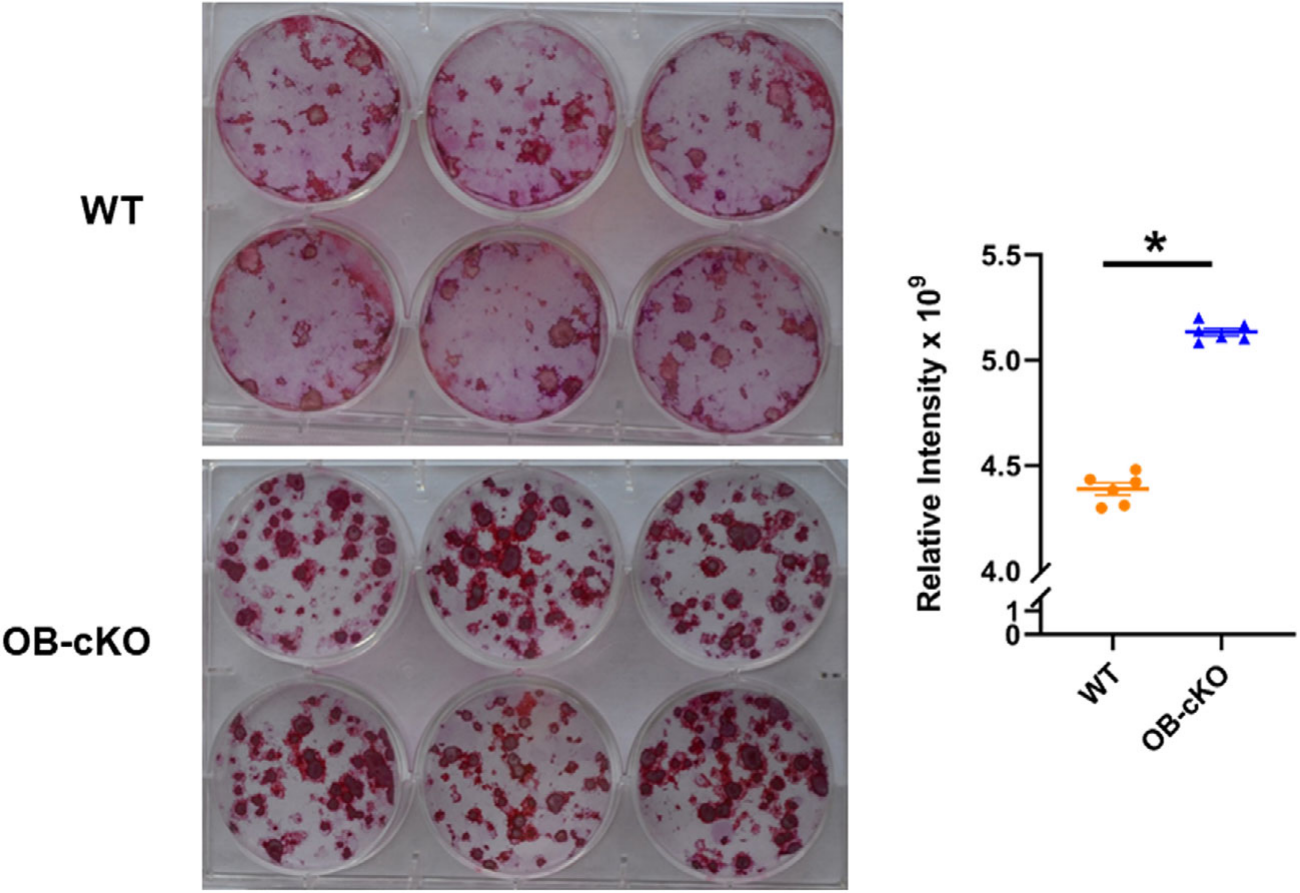
Mineralization was increased in osteoblasts from conditional knockout mice with an osteoblast‐specific deletion of OGR1 (OB‐cKO) compared with wild‐type (WT) osteoblasts. Bone marrow stromal cells (BMSCs) from OB‐cKO and WT mice were differentiated to osteoblasts and cultured for 21 days, then fixed and stained with alizarin red (left panel). Quantitation is shown on the right. Results are mean ± SE for 6 plates/group. * *p* < 0.05 versus WT.

**Fig. 4:**
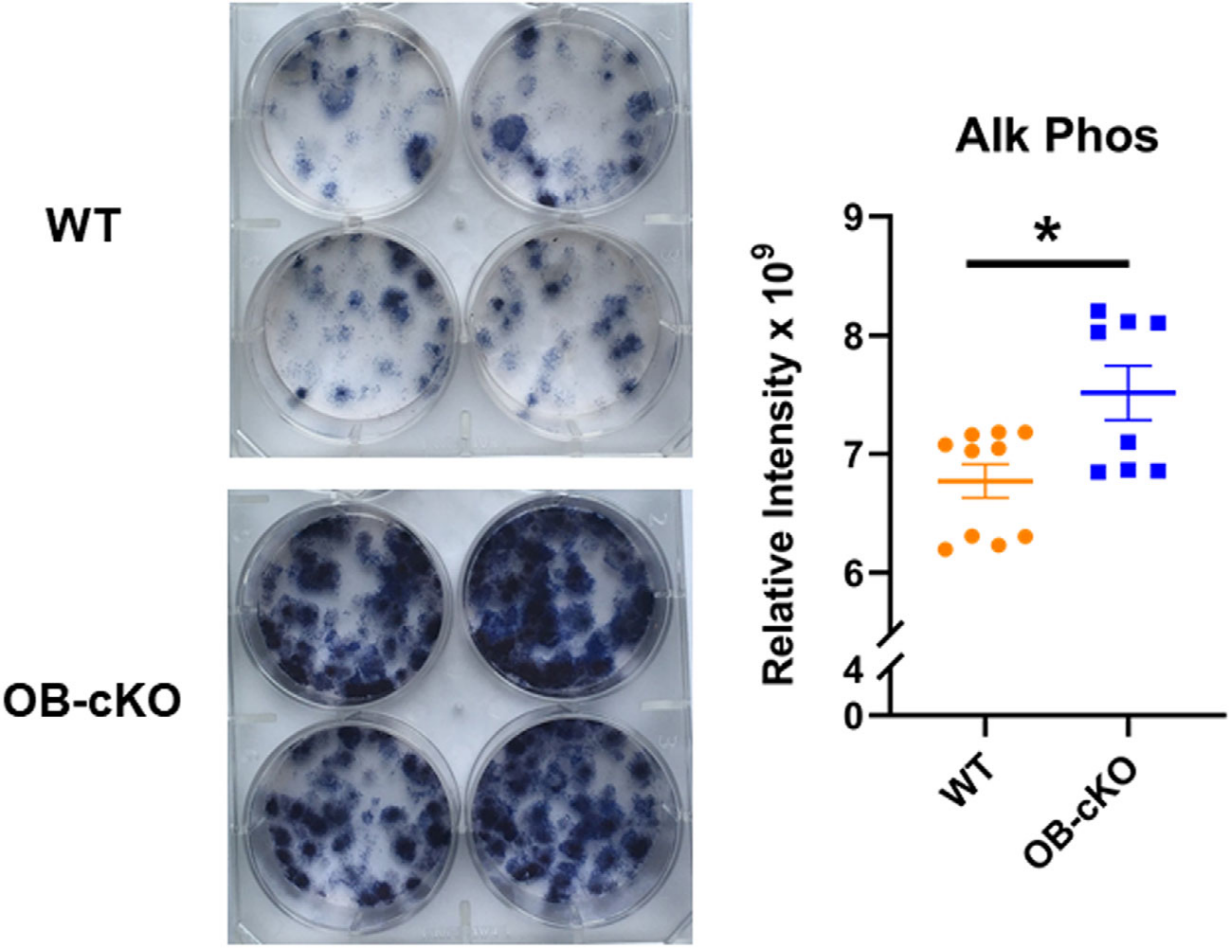
Alkaline phosphatase staining of differentiated osteoblasts was increased in conditional knockout mice with an osteoblast‐specific deletion of OGR1 (OB‐cKO) compared with wild‐type (WT) cells. Differentiated bone marrow stromal cells (BMSCs) were cultured for 14 days, fixed, and stained for alkaline phosphatase (left panel). Quantitation is shown on the right. Results are mean ± SE for 8–10 plates/group. *p < 0.05 versus WT.

BMSC from OB‐cKO and WT mice were cultured in differentiation medium for 3 weeks and the resultant OB cultures were then stained with alizarin red to measure their ability to mineralize. There was significantly more staining observed with the OB‐cKO cells, indicating greater mineralization in the absence of OGR1 compared with WT osteoblasts (Fig. 3). Similarly, OB‐cKO cells cultured for 2 weeks in differentiation medium showed greater alkaline phosphatase staining compared with WT osteoblasts (Fig. 4), further supporting increased mineralization in the absence of OGR1 in osteoblasts.

**Fig. 5:**
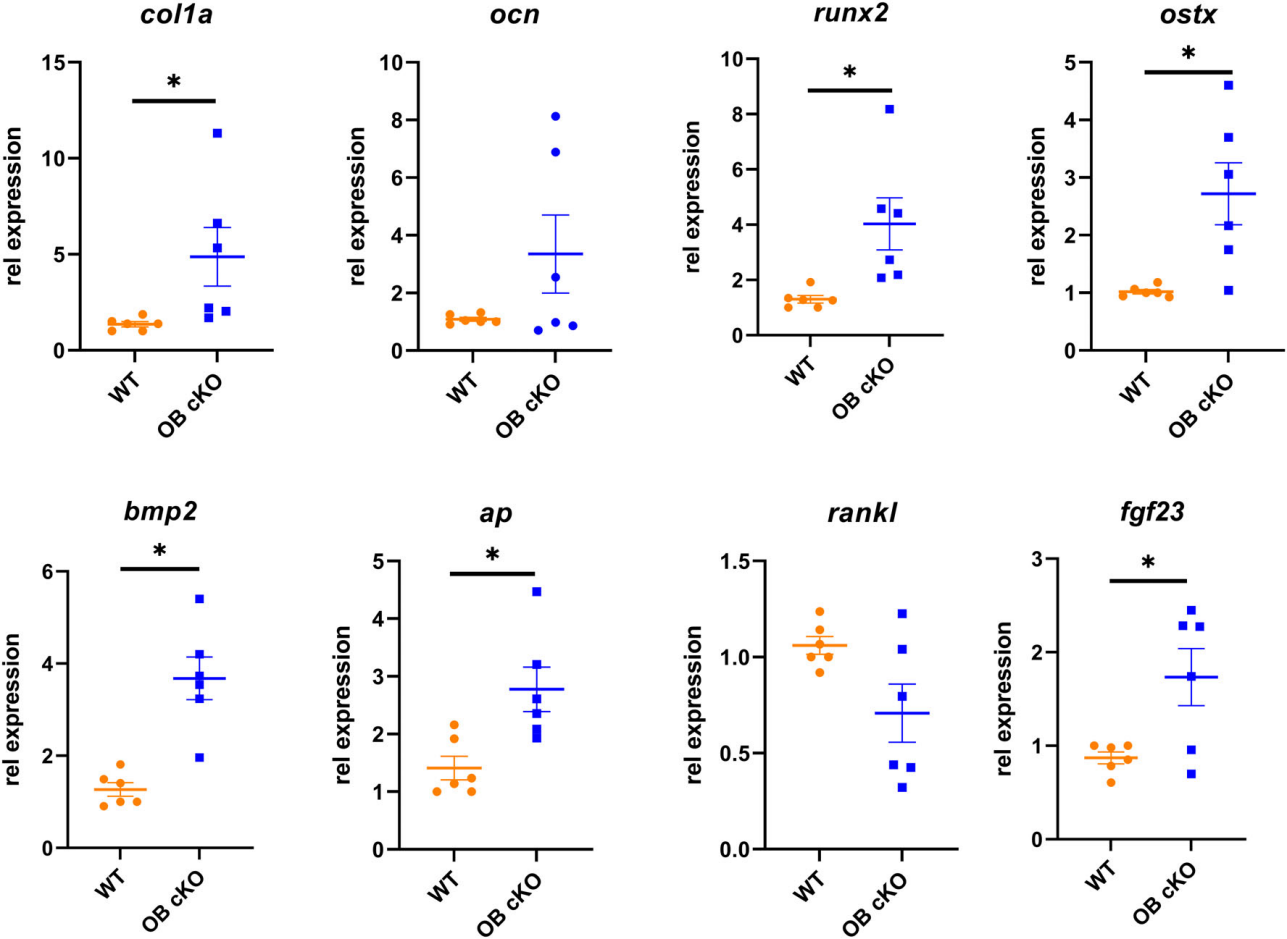
Osteoblastic gene expression was increased in bone marrow stromal cells (BMSCs) from conditional knockout mice with an osteoblast‐specific deletion of OGR1 (OB‐cKO) compared with wild‐type (WT) mice differentiated to osteoblasts. Differentiated osteoblasts were cultured for 14 days and RNA collected. Specific expression of collagen 1a (*col1a*), osteocalcin (*ocn*), runt‐related transcription factor 2 (*runx2*), osterix (*ostx*), bone morphogenic protein 2 (*bmp2*), alkaline phosphatase (*ap*), receptor activator of NF‐κB ligand (*rankl*) and fibroblast growth factor 23 (*fgf23*) was determined. RPL13A was used as an internal control gene. Results are mean ± SE from 6 cultures each. *p < 0.05 versus WT.

RNA was collected from osteoblastic cells after 2 weeks in differentiation medium and analyzed for specific osteoblast gene expression. There were significant increases in expression of collagen 1a, runx2, osterix, bone morphogenic protein 2, alkaline phosphatase, and FGF23 in OB‐cKO osteoblasts compared with WT osteoblasts (Fig. 5). No differences in osteoblast gene expression were found in osteoblasts differentiated from OC‐cKO BMSC compared with WT osteoblasts (Supplemental Fig. S1).

### Characterization of osteoclasts from OB‐cKO mice

**Fig. 6:**
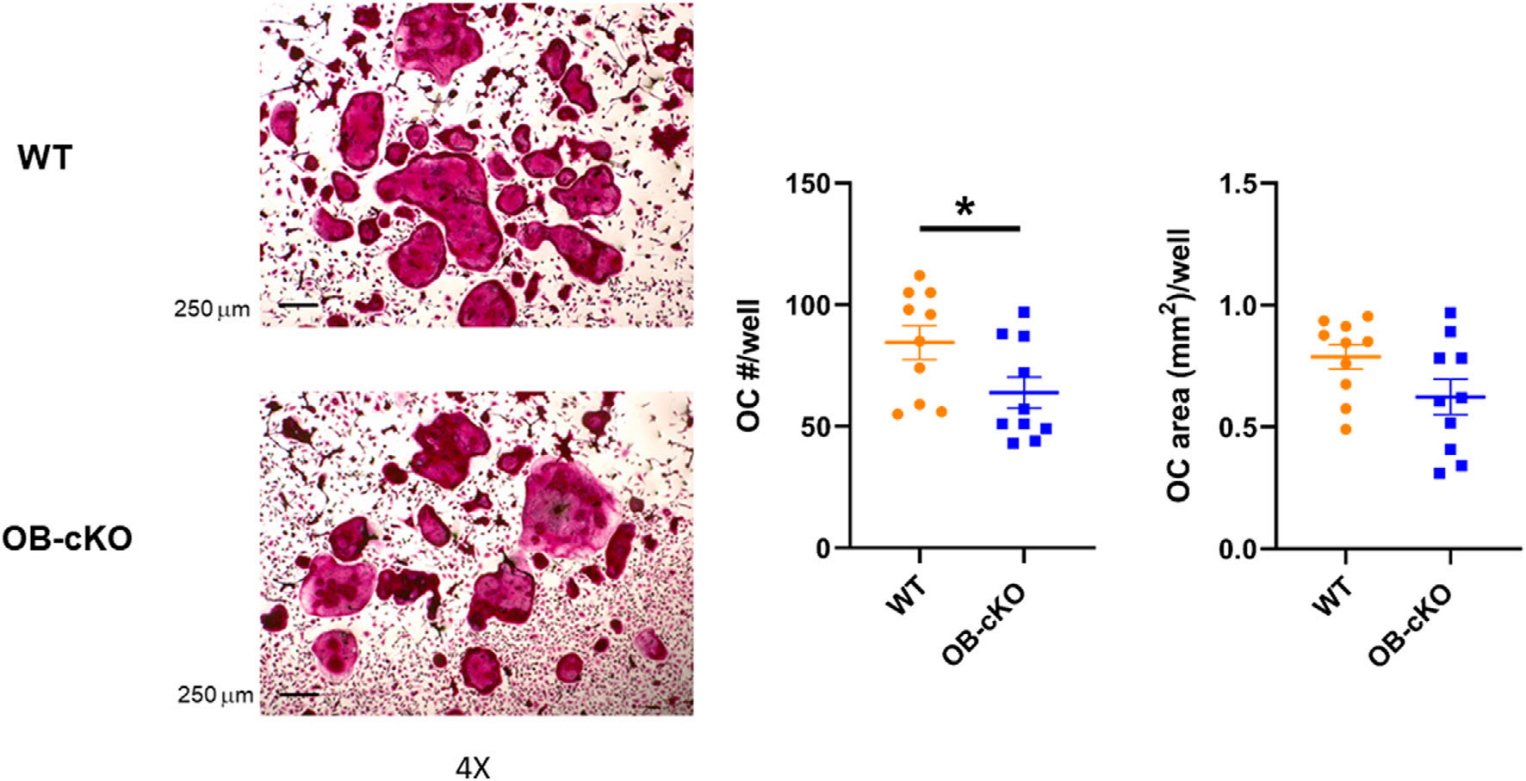
Tartrate‐resistant acid phosphatase (TRAP) staining of osteoclasts from conditional knockout mice with an osteoblast‐specific deletion of OGR1 (OB‐cKO) compared with wild‐type (WT) mice. Marrow cells from WT and OB‐cKO mice were cultured in 96‐well plates and differentiated to osteoclasts (OC) in the presence of macrophage colony‐stimulating factor (M‐CSF) and receptor activator of NF‐κB ligand (RANKL). These differentiated OC were then stained for TRAP (left panel). Osteoclast number/well and area/well were quantitated for mean ± SE for 10 wells/group (right panel). *p < 0.05 versus WT.

**Fig. 7:**
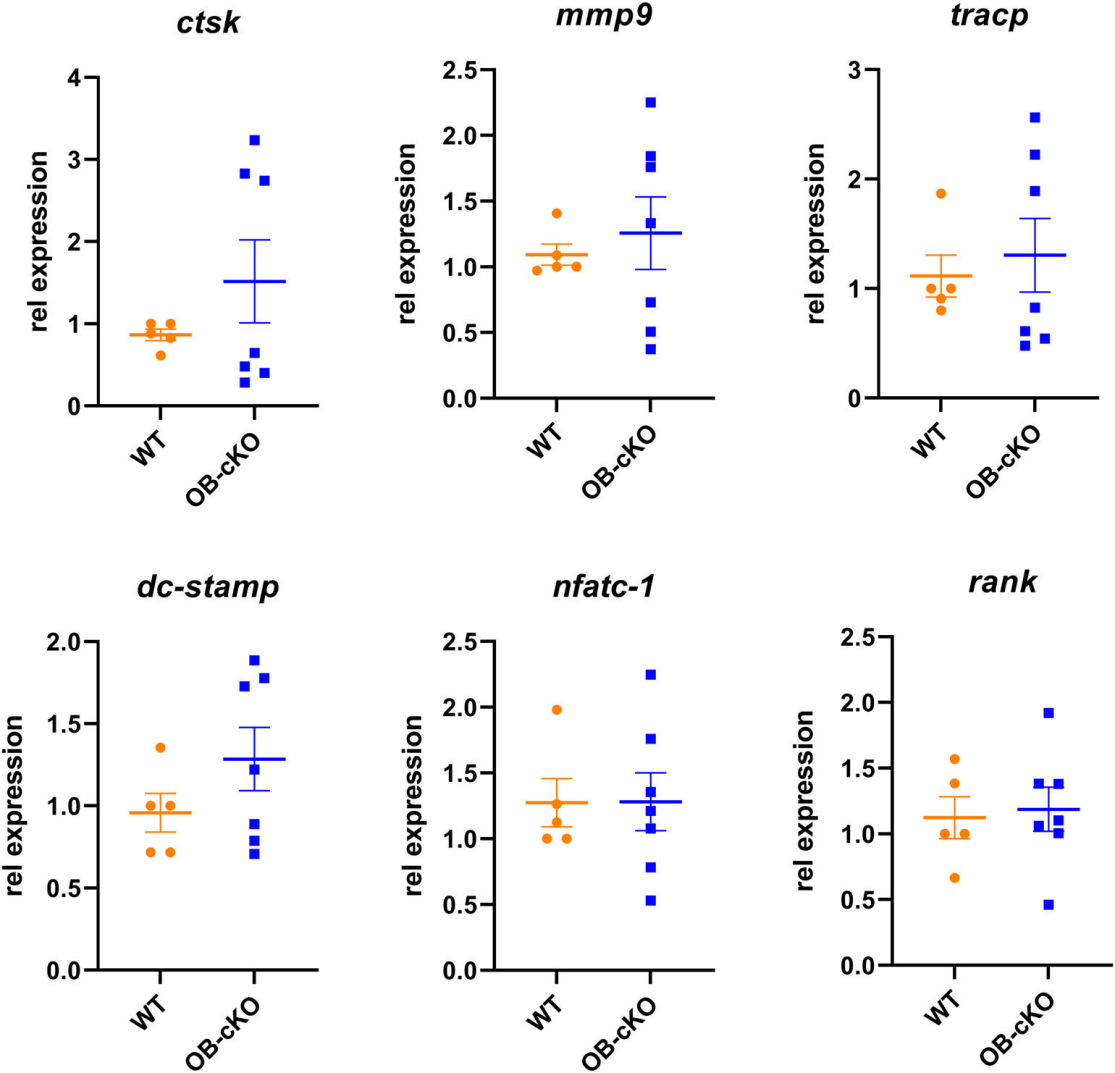
Osteoclastic gene expression from conditional knockout mice with an osteoblast‐specific deletion of OGR1 (OB‐cKO) compared with wild‐type (WT) mice. Marrow cells were cultured in 100 mm dishes and differentiated to osteoclasts (OC) in the presence of macrophage colony‐stimulating factor (M‐CSF) and receptor activator of NF‐κB ligand (RANKL) for 4 days. RNA was then collected from the differentiated osteoclasts and analyzed for osteoclast‐specific gene expression, including cathepsin K (*ctsk*), matrix metalloproteinase 9 (*mmp9*), tartrate‐resistant acid phosphatase (*trap*), dendritic cell‐specific transmembrane protein (*dc‐stamp*), nuclear factor of activated T cells 1 (*nfatc‐1*), and receptor activator of NF‐κB (*rank*). RPL13a was used as an internal control gene. Results are mean ± SE for 5–7 cultures each.

Marrow stromal cells from OB‐cKO mice were differentiated to osteoclasts in the presence of M‐CSF and RANKL and then stained with TRAP to demonstrate activated osteoclasts. There was a significant decrease in the number of stained osteoclasts from OB‐cKO mice compared with WT but no difference in osteoclast area (Fig. 6*A*, *B*). In other experiments, RNA was isolated from differentiated osteoclasts cultured from OB‐cKO and WT marrow cells and analyzed for osteoclast‐specific gene expression. No differences in osteoclast gene expression were observed for OB‐cKO compared with WT osteoclasts (Fig. 7).

### Response of cultured osteoblasts to metabolic acidosis

**Fig. 8:**
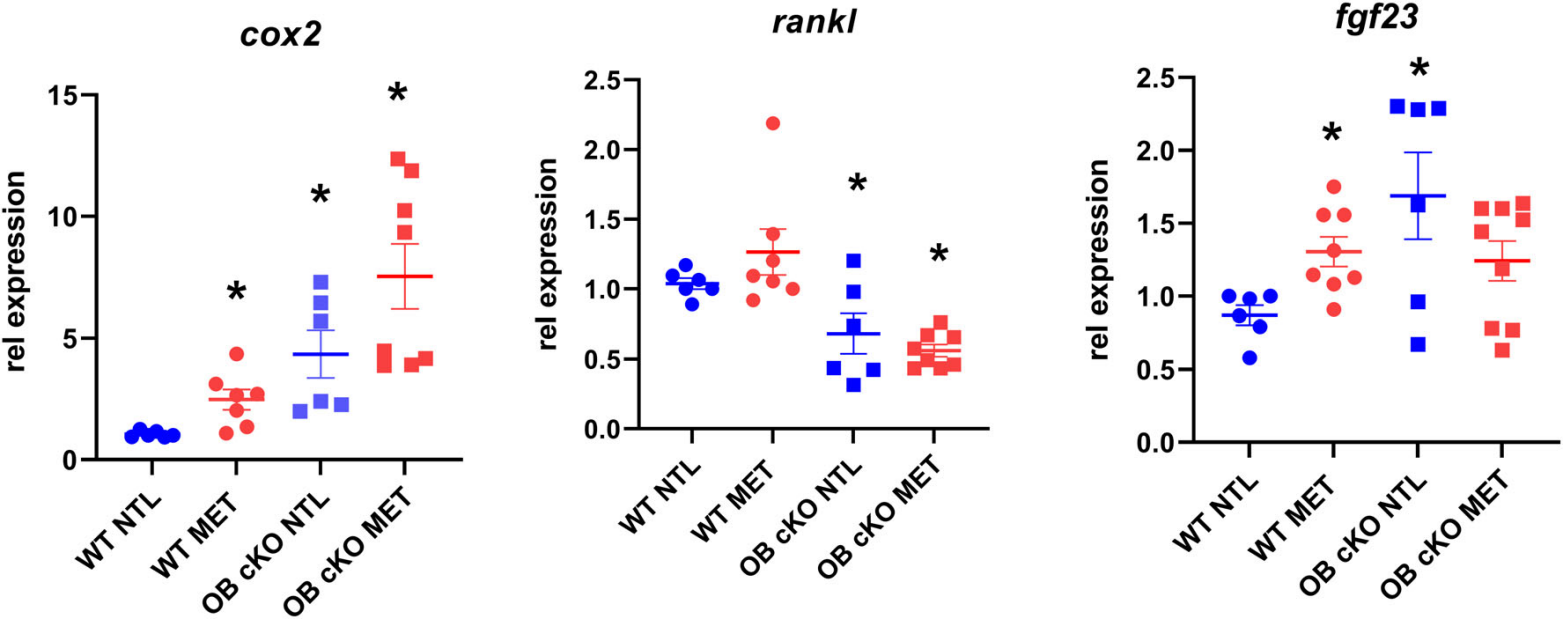
Response of osteoblasts from conditional knockout mice with an osteoblast‐specific deletion of OGR1 (OB‐cKO) and wild‐type (WT) mice to metabolic acidosis. Bone marrow stromal cells (BMSCs) from OB‐cKO and WT mice were differentiated to osteoblasts and cultured for 2 weeks. Medium was then changed to medium pre‐equilibrated to pH = 7.45 (neutral [NTL]) or pH = 7.15 (metabolic acidosis [MET]) and the incubation continued for 6 hours. Cells were then collected and RNA analyzed for specific gene expression of cyclooxygenase 2 (*cox2*), receptor activator of NF‐κB ligand (*rankl*), and fibroblast growth factor 23 (*fgf23*). RPL13a was used as an internal control gene. Results are the mean ± SE for 6–8 cultures/group. *p < 0.05 versus WT NTL group.

To determine whether there was a difference in the responsiveness of OB‐cKO osteoblasts to a physiologic reduction in pH, beyond the baseline differences with WT osteoblasts characterized above, differentiated osteoblasts were cultured for 6 hours in physiological CO_2_/HCO_3_^−^ buffered neutral (pH = 7.45) or acid (pH = 7.15) medium. There was a significant stimulation of cyclooxygenase 2 (*cox2*) and fibroblast growth factor 23 (*fgf23*) in response to MET in WT osteoblasts. Baseline gene expression of *cox2* and *fgf23* was increased in OB‐cKO cells compared with WT cells, but no significant stimulation of gene expression was observed in OB‐cKO osteoblasts in response to MET (Fig. 8). No change was observed for *rankl* in response to acid, although the baseline expression of *rankl* was significantly decreased in OB‐cKO osteoblasts compared with WT.

## Discussion

OGR1 has been found in multiple tissues and functions as a proton sensor in a variety of circumstances.^(^ ^16^, ^17^ ^)^ We and others have previously demonstrated the presence of OGR1 in osteoblasts^(^ ^8^, ^18^, ^19^ ^)^ and osteoclasts.^(^ ^22^, ^23^, ^24^, ^25^, ^26^ ^)^ Our previous results in calvariae and primary osteoblasts from CD‐1 mice suggested that OGR1 in the osteoblast mediated the resorptive effect of MET. We have shown that this G protein‐coupled proton receptor is required for the response of bone to MET. Initial activation of OGR1 by MET in the osteoblast leads to an increase in intracellular Ca signaling^(^ ^8^ ^)^ and subsequent stimulation of prostaglandin E_2_ synthesis, resulting in stimulation of osteoclastic bone resorption in neonatal mouse calvariae in culture.^(^ ^10^, ^31^ ^)^ Tomura and colleagues also found that OGR1 transduces the signal for acid in human osteoblasts via prostaglandin E_2_.^(^ ^19^ ^)^ In mice, we found that MET regulates both osteoblast and osteoclast activity, leading to a decrease in collagen synthesis and subsequent mineralization and an increase in osteoclastic bone resorption. There is communication between the osteoblast and osteoclast, but there are also direct effects of MET on osteoclasts.

To characterize the role of OGR1 in MET stimulation of bone resorption, we initially examined mice with a global deletion of OGR1.^(^ ^20^ ^)^ In the absence of OGR1, these mice had increased bone density and increased osteoblastic activity; however, there was also histologic evidence for increased osteoclast activity in these rapidly growing mice in vivo. As OGR1 is present in both osteoblasts and osteoclasts, it was not clear which cell type was critical for the OGR1‐mediated stimulation of bone resorption by MET. Although we previously found direct OGR1‐mediated effects of MET in primary osteoblasts,^(^ ^8^, ^10^ ^)^ we recently examined a role for OGR1 in the osteoclast using an osteoclast‐specific deletion (OC‐cKO) of OGR1.^(^ ^26^ ^)^ The OC‐cKO mice demonstrated increased bone density compared with WT mice, similar to the global OGR1 deletion mice. In contrast to the global knockout mice, TRAP staining of bones from the OC‐cKO mice showed a decrease in osteoclast number and size and isolated osteoclasts differentiated from marrow cells were also decreased in number and size compared with WT. Osteoclast‐specific RNA expression was decreased, along with osteoclast‐specific responses to MET when differentiated osteoclasts from OC‐cKO marrow were compared with WT osteoclasts.

To determine the relative importance of MET regulation of OGR1 in the osteoblast, the current study examines the changes observed as a result of osteoblast‐specific deletion of OGR1. Mice with an osteoblast‐specific deletion of OGR1 were generated by successful breeding of OGR1 flox/flox mice with Col1a1‐Cre mice. Immunofluorescent staining of osteoblasts differentiated from BMSC demonstrated the OGR1 deletion specifically in the OB‐cKO osteoblasts. Genotyping showed the presence of the mutant OGR1 in OB‐cKO osteoblasts and not in WT osteoblasts or in osteoclasts from OB‐cKO mice, demonstrating the specificity of the OGR1 deletion. OB‐cKO mice had increased cortical bone area, though there was no change in trabecular bone parameters. It is possible that using a different Cre‐promoter targeting an earlier stage in osteoblast lineage could lead to a greater effect on bone phenotype. However, significant differences in the baseline characteristics of differentiated osteoblasts were found in cells from the OB‐cKO mice. Increased mineralization as evidenced by alizarin red staining and increased alkaline phosphatase staining were observed in OB‐cKO osteoblasts compared with WT osteoblasts. Increased expression of osteoblast‐specific genes was also observed in OB‐cKO osteoblasts, whereas there were no differences observed in osteoblasts from OC‐cKO mice compared with WT osteoblasts. The changes in osteoblast gene expression were comparable to what was observed in the global OGR1 knockout mice. Unlike what has been found for OC‐cKO osteoclasts, there was no difference in osteoclast‐specific gene expression in osteoclasts from OB‐cKO mice compared with WT osteoclasts. There was a small effect to decrease the number of osteoclasts, but not the size of osteoclasts, in the OB‐cKO mice compared with WT osteoclasts. This is in contrast to the significant decrease in both number and area observed in osteoclasts from OC‐cKO mice.^(^ ^26^ ^)^

Our initial study using the global OGR1 knockout mice was carried out in male mice, whereas this study was performed using female mice. We chose to study female mice because in our characterization of the osteoclast‐specific OGR1 deletion, there was a significantly greater effect on bone phenotype in females compared with males. In the OB‐cKO mice, there was only a small change in the bone of both females and males, but to do direct cellular comparisons with the OC‐cKO mice, it was important also to use female mice for the current study. Given that the increase in osteoblast‐specific gene expression found for the female OB‐cKO osteoblasts compared with WT was very comparable to the increase in osteoblast gene expression found in the male global OGR1 knockout osteoblasts, the sex differences may not be important. However, it has been reported that a large number of individual gene knockouts have resulted in dimorphism based on sex as well as differential effects on long bones and vertebral bones.^(^ ^33^ ^)^ Others have also found that specific gene knockouts exhibit sex‐related differences in phenotypes,^(^ ^33^, ^34^, ^35^, ^36^, ^37^ ^)^ including a vitamin D receptor knockout that showed differences in skeletal phenotype.^(^ ^38^ ^)^ Sexual dimorphism is present in many mammalian phenotypic traits^(^ ^39^ ^)^ and sex‐chromosome dosage effects on gene expression have been described, although we do not yet know if any of this pertains to our results. It is possible that gene deletions themselves could have effects not yet considered.

To examine whether there was any change in responsiveness of OB‐cKO osteoblasts to MET stimulation, we tested changes in expression of several genes that have been shown to respond to MET in WT osteoblasts. Deletion of OGR1 eliminated the MET‐induced stimulation of *Cox‐2* and *Fgf23* observed in WT osteoblasts after 6 hours in acidic medium, similarly to what we have observed in primary mouse calvarial osteoblasts.^(^ ^11^, ^40^ ^)^ No difference in *rankl* expression in response to MET was observed after 6 hours, but previous studies have suggested a MET‐stimulation of *rankl* expression may require up to 48 hours to be evident.^(^ ^10^, ^12^ ^)^

Although there are fewer reports that consider the role of OGR1 in the osteoblast compared with the osteoclast, our studies of selective deletion of OGR1 suggest that the receptor in both osteoblasts and osteoclasts is important for regulation of MET‐induced bone resorption. There were clear increases in baseline osteoblast activity as well as a decrease in MET‐induced stimulation of *cox2* and *fgf23* gene expression in differentiated osteoblasts from OB‐cKO mice in the absence of any changes in OGR1 in the osteoclasts of these mice. There is communication between osteoblasts and osteoclasts during bone remodeling, for example, with RANKL produced in the osteoblast stimulating osteoclast activation and differentiation,^(^ ^41^, ^42^, ^43^ ^)^ but it seems that direct MET activation of OGR1 in each of these cell types is necessary for the full response of bone to acidosis. Future studies will be critical to further define the importance of the sexual dimorphism that has been observed for the effects of deletion of either the OB or OC‐specific OGR1. The current study examined only the deletion of OGR1 from mature osteoblasts. Using CRE‐recombinase coupled to promoters for earlier stages of osteoblast differentiation would further expand our understanding of the role of OGR1 in bone formation and its response to MET.

These OB‐ and OC‐specific OGR1 conditional knockout models provide an additional tool to map the important signaling pathways initiated by acidosis to stimulate bone resorption. Patients with chronic kidney disease and those on dialysis are less able to excrete acid, develop metabolic acidosis, and have significant abnormalities in bone quality, leading to increased risk of fracture.^(^ ^44^, ^45^ ^)^ Better understanding of the mechanism of MET‐induced bone resorption could ultimately provide new approaches for decreasing the bone loss associated with chronic metabolic acidosis in patients with chronic kidney disease.

## Disclosures

NSK reports grants from the Renal Research Institute and the National Institutes of Health (NIH) to support this research. She owns stock in Amgen and her spouse has stock and stock options from Tricida and stock in Amgen and is a consultant for Tricida, Sanofi Genzyme, and Relypsa/Vifor Fresenius. DAB reports grants from the NIH to support this research. He owns stock and stock options from Tricida and stock in Amgen and is a consultant for Tricida, Sanofi Genzyme, and Relypsa/Vifor Fresenius and adjudicates adverse events from Novo Nordisk/Covance (all outside the submitted work). All other authors have no competing interests.

## Author Contributions

**Nancy S. Krieger:** Conceptualization; data curation; formal analysis; funding acquisition; investigation; methodology; project administration; supervision; visualization; writing – original draft; writing – review and editing. **Luojing Chen:** Data curation; investigation; methodology; data analysis, writing – review and editing. **Jennifer Becker:** Investigation; methodology; writing – review and editing. **Michaela Chan:** Investigation; methodology; writing – review and editing. **David A. Bushinsky:** Funding acquisition; resources; writing – review and editing.

### Peer Review

The peer review history for this article is available at https://publons.com/publon/10.1002/jbm4.10691.

## Supporting information


**Supplemental Fig. S1.** No difference in osteoblast‐specific gene expression in osteoblasts.

## Data Availability

The data that support the findings of this study are available from the corresponding author upon reasonable request.
