# Supplementary figures and images for: Effect of Osteoblast‐Specific Deletion of the Proton Receptor OGR1
Source: JBMR Plus. 2022 Oct 31;6(12):e10691. doi: 10.1002/jbm4.10691 (PMC9751651; doi:10.1002/jbm4.10691)

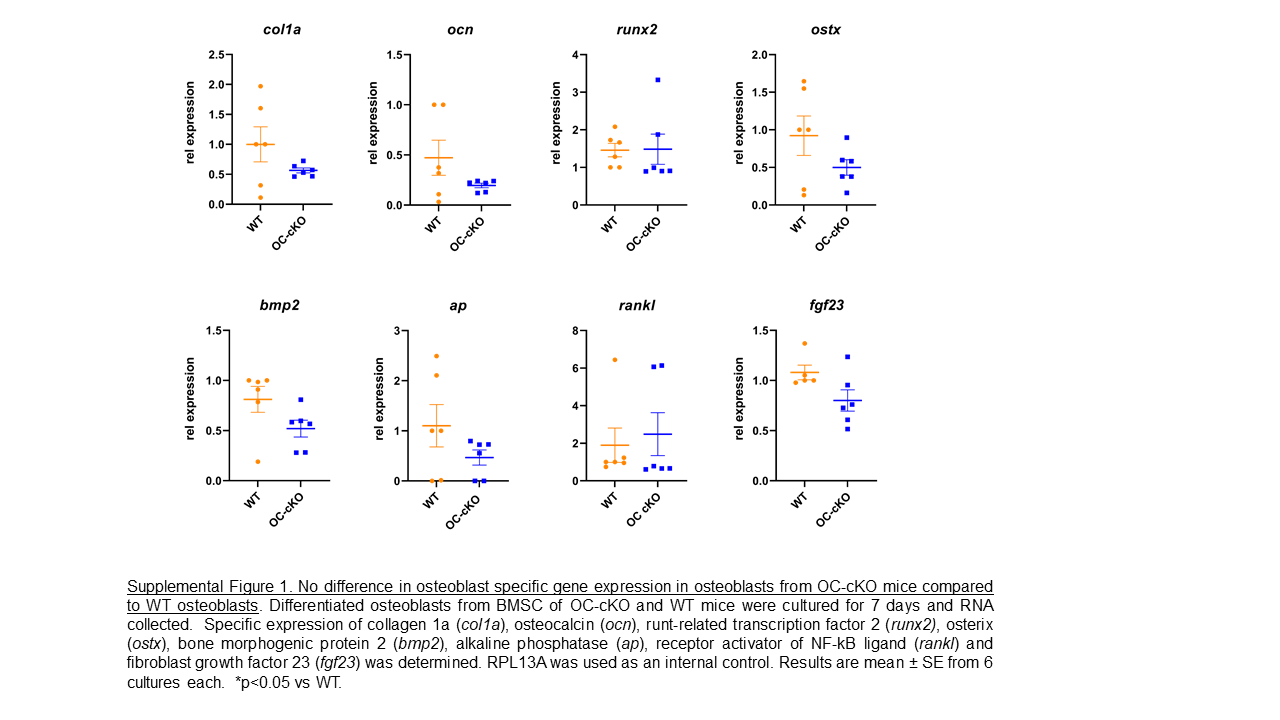

Supplement: Supplementary file 1 — Supplemental Fig. S1. No difference in osteoblast‐specific gene expression in osteoblasts. [file JBM4-6-e10691-s001.tif]
